# Testosterone Affects Song Modulation during Simulated Territorial Intrusions in Male Black Redstarts (*Phoenicurus ochruros*)

**DOI:** 10.1371/journal.pone.0052009

**Published:** 2012-12-17

**Authors:** Beate Apfelbeck, Sarah Kiefer, Kim G. Mortega, Wolfgang Goymann, Silke Kipper

**Affiliations:** 1 Abteilung für Verhaltensneurobiologie, Max-Planck-Institut für Ornithologie, Seewiesen, Germany; 2 AG Verhaltensbiologie, Institut für Biologie, Freie Universität Berlin, Berlin, Germany; 3 Abteilung für Ornithologie, Universität Konstanz, Konstanz, Germany; Pennsylvania State University, United States of America

## Abstract

Although it has been suggested that testosterone plays an important role in resource allocation for competitive behavior, details of the interplay between testosterone, territorial aggression and signal plasticity are largely unknown. Therefore, we investigated if testosterone acts specifically on signals that communicate the motivation or ability of individuals to engage in competitive situations in a natural context. We studied the black redstart, a territorial songbird species, during two different life-cycle stages, the early breeding phase in spring and the non-breeding phase in fall. Male territory holders were implanted with the androgen receptor blocker flutamide (Flut) and the aromatase inhibitor letrozole (Let) to inhibit the action of testosterone and its estrogenic metabolites. Controls received a placebo treatment. Three days after implantation birds were challenged with a simulated territorial intrusion (STI). Song was recorded before, during and after the challenge. In spring, both treatment groups increased the number of elements sung in parts of their song in response to the STI. However, Flut/Let-implanted males reacted to the STI with a decreased maximum acoustic frequency of one song part, while placebo-implanted males did not. Instead, placebo-implanted males sang the atonal part of their song with a broader frequency range. Furthermore, placebo-, but not Flut/Let-implanted males, sang shorter songs with shorter pauses between parts in the STIs. During simulated intrusions in fall, when testosterone levels are naturally low in this species, males of both treatment groups sang similar to Flut/Let-implanted males during breeding. The results suggest that song sung during a territorial encounter is of higher competitive value than song sung in an undisturbed situation and may, therefore, convey information about the motivation or quality of the territory holder. We conclude that testosterone facilitates context-dependent changes in song structures that may be honest signals of male quality in black redstarts.

## Introduction

Sexually selected signals often serve both to attract a mate and to advertise competitive abilities, for example during territorial disputes (reviewed in [1]). Studying the song of male passerine birds may advance our understanding of the mechanisms underlying the use and coordination of such signals. A range of studies revealed that song characteristics can transfer information about the quality of the singer (reviewed in [2]) such as its immune system [3], age (reviewed in [4]), early experience [5] or motivation to contribute to breeding [6].

In many species males modulate their song in an aggressive context: they might select certain song types matching a rival [7], or produce specific song elements only in situations of high arousal [8]. In addition, birds can change song characteristics such as frequency patterns and trill rate [9], [10]. Male as well as female listeners respond differentiated to such modulations [11]–[14]. Song modulations can occur on two domains: on the one hand, birds may change the general output of song (e.g. song rate or amplitude), i.e. measures that potentially every male can vary within broad limits. On the other hand, modulation also occurs in structural song characteristics. Structural characteristics describe, for example, song repertoire characteristics [15] or song parts that are challenging to sing, such as rapid broadband trills (reviewed in [16]), specific song trills [17] or consistent syllables [18]. Structural song patterns have been classified as ‘index signals’ that honestly communicate a physical trait related to male quality [19]. Only very few studies have revealed a capability of individuals to modulate such physically constrained signals within narrow limits [9], [10], [20], [21]. Thus, from a functional point of view, index signals such as structural song parameters should play an important role in the communication of competitive ability.

The steroid hormone testosterone plays an important role in the regulation of adult singing and territorial behaviors and the associated vocalizations during breeding are facilitated by testosterone in a wide range of male vertebrates (reviewed in [22], [23]). Therefore, it has been suggested that testosterone might play an important role in resource allocation for competitive behavior during reproduction (reviewed in [24]). From this point of view, testosterone should act specifically on signals that communicate the motivation or ability of individuals to engage in competitive situations and is, therefore, expected to be involved in context-dependent adjustment of such signals. However, details of the interplay between hormones, territorial aggression and signal plasticity in a natural context are largely unknown.

Manipulations of testosterone levels may alter song output (measured, for example, as song rate or duration; e.g. [25]–[29]). Whether testosterone also affects structural song parameters is less clear. In barn swallows (*Hirundo rustica*), the duration and pulse rate of the harsh ‘rattle’ element correlated moderately with absolute testosterone levels [30]. Manipulation studies suggested that zebra finches (*Taeniopygia guttata*) treated with testosterone decreased the fundamental frequency of harmonic stacks in their song [31]. Other correlational and experimental studies with testosterone treatment failed to find effects on structural song parameters [29], [32], [33]. Studies that implant birds with testosterone may be problematic, because especially immediately after implantation testosterone may circulate in pharmacological doses [34], [35]. It is thus questionable whether manipulations exclusively within the physiological range of testosterone would reveal similar results. Treatments inhibiting the action of testosterone or its major metabolite estradiol by blocking the androgen receptor and/or the conversion to estradiol avoid such pharmacological effects (but can only inhibit, not enhance effects of steroid hormones). The – so far - only study in which the androgenic and estrogenic pathways of testosterone action were blocked failed to find effects on structural song parameters in great tits (*Parus major*, [36]). Thus, to the best of our knowledge, an effect of physiological changes in testosterone on structural measures of song has not been demonstrated so far.

Song plasticity and its potentially underlying hormonal mechanisms may be studied in different contexts, such as during spontaneous singing or singing during a territorial challenge because the song used (output and structure), the information transmitted (e.g. quality and/or competitive ability) and the receiver and/or audience (other males and/or females) may differ in these contexts. Thus, depending on context, song may be facilitated by sex steroids or not. Furthermore, several songbird species also sing outside the breeding season, providing an additional contextual variable. Song characteristics (of spontaneous song) differ between the breeding – and non-breeding season: For example, some species produce more repetitive elements [15], [37]–[39], longer songs [40] and more stereotypic song [37] in spring than in fall. Comparisons between breeding and non-breeding song were so far restricted to spontaneously produced song. Whether changes in song during a territorial challenge also differ between seasons has not been studied yet. Such a difference should be expected from a functional point of view, since fall territories are not of immediate importance for reproduction. Those seasonal differences in song might well be mediated by testosterone levels, because in most songbird species testosterone levels are low during non-breeding [41]–[44]. It remains open, however, whether and how testosterone is involved in context-dependent song plasticity during the non-breeding season (e.g. [45]). In song sparrows, for example, testosterone also regulates territorial behaviour during the non-breeding season, presumably through steroids of non-gonadal origin that are then metabolized to testosterone and estradiol directly in the brain [46].

In this study, we investigated the role of testosterone in regulating spontaneous song and song in an aggressive context in free-living male black redstarts (*Phoenicurus ochruros*). The species is well-suited to study this topic as there is evidence that song structures may contain information about the competitive ability or motivation [47]. Black redstarts show delayed song maturation, i.e. adult and yearling males differ in structural song parameters [47] such as the duration of song parts and number of elements or frequential song patterns, as well as in visual traits (delayed plumage maturation) [48]. These age-differences are also reflected in simulated territorial intrusions: adult and yearling males respond differentially to playbacks of the two age classes [47]. Despite this delayed maturation, adults as well as one year olds reproduce, but adult males usually occupy higher quality habitats and have a higher reproductive success [49]. Although this has not yet been studied, it seems plausible to assume that behavioral and morphological age-differences may be accompanied by different hormonal profiles.

Furthermore, black redstarts not only defend territories in spring after having returned from their wintering grounds, but also in fall, just after molt and before migration [50]. During the territorial phase in fall they have low testosterone levels [51].

Against this background, we tested the role of testosterone in the modulation of song characteristics in this species. We did so by implanting birds with an androgen receptor blocker (flutamide) and an aromatase inhibitor (letrozole) which inhibits the conversion of testosterone to estradiol, as testosterone can modulate behavior either directly by binding to androgen receptors or indirectly by conversion to estradiol and binding to estrogen receptors [52]. As controls we used birds treated with placebo implants. After implantation, we first recorded the spontaneous song of territorial males in an undisturbed context and then conducted a playback experiment simulating a territorial intrusion (STI) by a foreign male. This procedure was conducted in spring during the early breeding season, and again in fall during non-breeding, using a different set of birds.

The aim of our study was threefold. First, we wanted to investigate whether black redstarts change structural song parameters in an aggressive context, i.e. whether song parameters differ between a non-challenged context before the STI and during/after the STI. Based on prior studies on black redstart song and in particular on a playback-study on song and age ( [47], see above) we expected to find changes in song output measures and structural song characteristics. Index signals that honestly communicate a physical trait related to male quality [19] are good candidates here. Thus, we expected those structural song parameters to change in the agonistic context that have been shown to be characteristic for adult males song, that is the number of song elements and the frequency-range of song parts [47]. Specifically we would expect focal males to sing longer song parts with trills, higher frequencies and/or with broader frequency bandwidth during a territorial encounter than in an undisturbed situation.

Second, by blocking the actions of testosterone, we attempted to determine the role of this hormone in context-dependent vocal plasticity. If testosterone is playing a key role in the resource allocation for competitive behavior (e.g. [53]) during the breeding season in spring, we would expect flutamide/letrozole-treated males (thereafter termed Flut/Let males) to invest less in those behaviors and song patterns that are relevant in such situations than placebo-males. Thus, changes in song during territorial encounters (see above) should be less pronounced or absent in Flut/Let males in contrast to placebo treated males.

Thirdly, because testosterone levels are low in fall [51] males should not change song parameters in an aggressive context in fall and the treatment with flutamide and letrozole should have no effect on song. We therefore, compared song behavior in an undisturbed and an agonistic context in fall again between Flut/Let males and placebo males. We predicted that in fall Flut/Let- and placebo-implanted males should not differ in their vocal response to a simulated territorial intrusion.

## Materials and Methods

### Ethics Statement

All experimental procedures were approved by the Committee on the Ethics of Animal Experiments of the governmental authorities of Upper Bavaria (Permit Number: Az. 55.2-1-54-2531-151-08). All surgery was performed under local carprofen anesthesia, and all efforts were made to minimize suffering.

### Study Site and Subjects

Adult territorial male black redstarts were caught during spring (9–27 April 2009) and fall (22 September - 7 October 2009) in Upper Bavaria, Germany (N 47°, E 11°) with mealworm-baited ground traps. To avoid potential age-related differences in song and hormonal profile (see Introduction) we restricted our study to males being 2 yrs or older. Birds were lured to the traps by broadcasting playbacks of the species’ song of short duration (<2 min). We remotely muted the loudspeaker as soon as the territory owner approached the traps. Conspecific playback does not influence testosterone levels in territorial male black redstarts [51]. Males were implanted with either one placebo pellet (spring: n = 10, fall: n = 6) or two time release pellets (spring: n = 10, fall: n = 6, 21 day release: 1.5 mg per pellet; release rate 71 µg/day; Ø = 3.2 mm, Innovative Research of America, Sarasota, FL) containing the androgen receptor blocker flutamide (Sigma-Aldrich F-9397) and the aromatase inhibitor letrozole (Novartis; [54]), respectively. Each male was implanted only once, either in spring or fall. Implants were inserted subcutaneously on the back between the wings through a small incision in the skin that was sealed with tissue glue afterwards (Nexaband; World Precision Instruments).

Control and experimental groups did not differ significantly in body mass (spring: t = 1.52, df = 17.9, p = 0.15, fall: W = 34.5, n = 16, p = 0.8), length of the right tarsus (spring: t = −0.25, df = 12.5, p = 0.8, fall: W = 44, n = 16, p-value = 0.2), length of the right wing (t = 0.25, df = 14.2, p = 0.8, fall: W = 41, n = 16, p-value = 0.4) and cloacal protuberance (CP) volume (spring: t = −0.17, df = 13.1, p = 0.9), which was estimated by calculating the volume of a cylinder [V = π*(CP width/2)2*CP height].

Each male was banded with a numbered aluminum ring (Vogelwarte Radolfzell) and a unique combination of three color rings for individual recognition. Measuring, ringing and implanting the birds took no longer than 25 min after which the males were released onto their territories.

### Experimental Design

Simulated territorial intrusions were conducted three days after implantation. All experiments were conducted between 8∶00 and 12∶00 h. For the playback we used spontaneous songs from 20 adult male black redstarts recorded in spring 2009 and from 12 adult males recorded in fall 2009 with the same equipment as reported below. For each target male we selected a playback that was recorded at least 10 km away from the study area. This distance was sufficient to avoid that target birds might know the stimulus birds. Playbacks were put together using Avisoft-SASLab Pro software, version 4.51 (Raimund Specht, Berlin, Germany). Each playback consisted of 20 songs recorded from one male. Songs were filtered (1 kHz high-pass filter) and amplitude was normalized to 75%. A playback consisted of each of two different song types (X and Y) played back in a XXYYXXYYXX sequence, with X and Y in 10 different versions (i.e. different exemplars of the same type). Songs were divided by pauses of 4.5 s. By repeating each sequence six times the playback had a duration of 20 minutes in total. This design resembled the organization of natural song in this species.

We presented each of the playbacks twice: once to a Flut/Let bird and once to a control bird, thereby alternating the presentation order between the two groups. These pairs of groups were tested in close temporal proximity in order to rule out e.g. seasonal or male status effects. In addition, by this paired design we were able to control for effects of different source bird song. The simulated territorial intrusion (STI) experiments were performed by placing a stuffed decoy (male in full adult plumage protected by an inconspicuous cage, three different decoys used) into the center of the respective territories. A remote-controlled loudspeaker (Foxpro Scorpion, digital game caller, FOXPRO Inc. Lewistown, USA) was put underneath the decoy to play back the territorial song of a potential rival at a sound pressure level of 65 dB SPL at 1 m (as measured with a CEL 573.B1 Sound Level Analyzer). We only started an experiment when a male was singing. The song was recorded 10 min prior to the start of the STI, during the 20 min STI and 10 min after the STI with a Sennheiser directional microphone (ME66/K6) connected to a Marantz solid state recorder PMD 660 (sampling frequency: 44.1 kHz; resolution: 16 bit).

### Data Analysis

**Figure 1:**
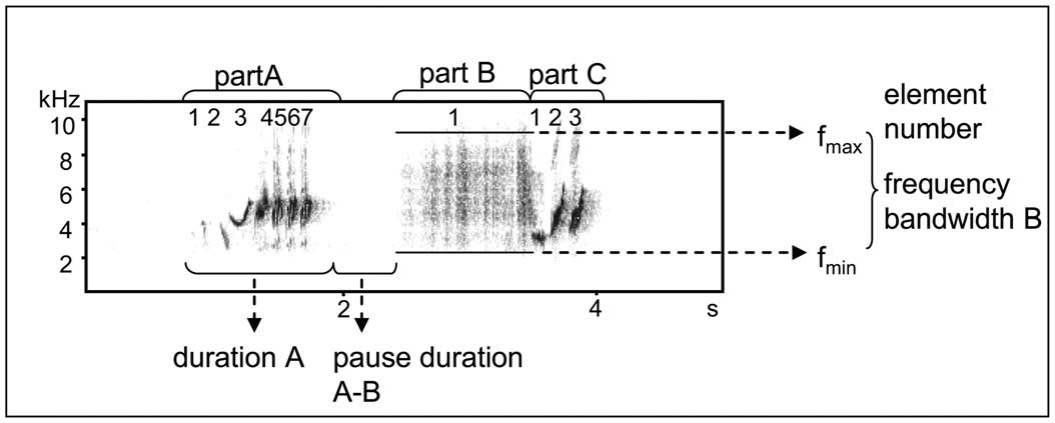
A song of a black redstart illustrating the acoustic measures analyzed (Spectrogram: Avisoft-SASLab Pro, sample rate 22, 050 Hz, FFT = 256 points, Hamming-Window, Overlap: 50%).

The song was analyzed using Avisoft-SASLab Pro software, version 4.51. Recordings were visualized in spectrograms (settings: sample rate 22,050 Hz, FFT = 256 points, Hamming-window, overlap: 50%). We determined the number of songs by visual inspection and selected songs of sufficient quality (low background noise) for further sound analysis. Each song of black redstarts can be divided into three distinct parts (part A, B and C, see Fig. 1 and e.g. [47]) with a pause of varying duration between part A and B. We measured the duration of parts A, B and C, the total song and the duration of pauses between A and B (Fig. 1). We counted the number of elements of part A and C (mean of max. 20 song renditions). We also determined the frequency bandwidth and the maximum frequency of part A, B and C using the automatic parameter measurement function (threshold −20 dB) in Avisoft (mean of max. 10 renditions of high-quality songs).

Data were analyzed with R version 2.9.1 [55]. Song before, during, and after the STI was analyzed using general linear mixed models with bird identity as a random effect to control for repeated measures. We analyzed whether the dependent variables (number of songs, song duration, duration of part A, B, C and the pause between part A and B, the number of elements in part A and C and maximum frequency and bandwidth of all parts) were influenced by the Flut/Let treatment, the context (testing phase of the STI) and their interaction. In all cases, dependent variables where transformed if assumptions of normality and/or equality of variances were not met. Significance was accepted at α ≤0.05 (two-tailed).

## Results

### Song in Spring

**Figure 2:**
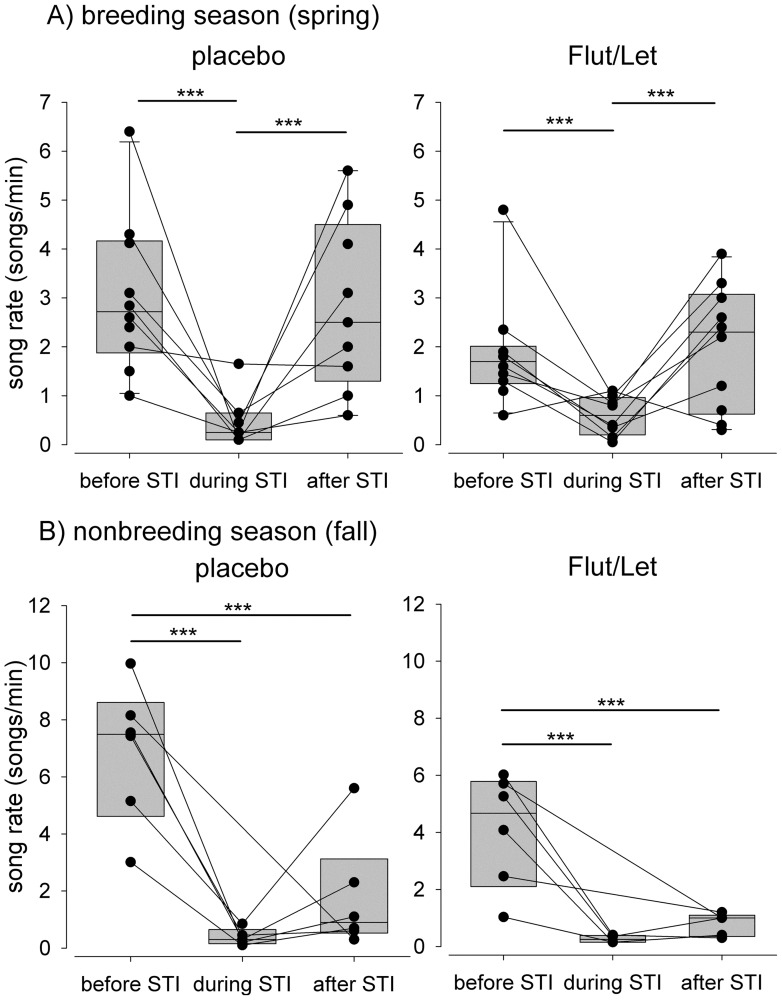
Song rate before, during and after the STI. Depicted separately for males treated with flutamide and letrozole (‘Flut/Let’) and placebo treated males (‘placebo’) in A) spring (n = 10 per group) and B) in fall (n = 6 per group). Each circle represents one individual male and measurements of the same male are connected by a line. Asterisks indicate significance (*** p<0.001) and are according to a priori set contrasts (before vs. STI and before vs. after the STI). Mind the different scales in A and B.

Males sang significantly fewer songs during the STI than when singing spontaneously before and after the STI (Fig. 2a, Table 1). Song duration significantly changed in placebo-implanted males, with songs during the STI being shorter than before or after the STI. In Flut/Let-males, song duration remained constant before, during and after the STI (Table 1). The shortening of the song in placebo-implanted males was mainly due to a significantly shorter pause between song part A and B (Table 1), because the durations of the three song parts (A, B and C) did not differ significantly before, during and after the STI (Table 1).

**Table 1:** Linear mixed model results for the effects of context and Flut/Let-treatment on song output and structure in spring.

element	treatment	context	interaction	Coheńs d [95% CI]	
				placebo	Flut/Let
song rate	F_1,18_ = 2.3	**F**_2,30_ ** = 23.6**	F_2,30_ = 1.3	**2.0**	**1.4**
	p = 0.1	**p<0.0001**	p = 0.3	**[0.8, 3.2]**	**[0.4, 2.5]**
song duration	F_1,18_ = 0.2	**F**_2,30_ ** = 6.7**	**F**_2,30_ ** = 3.8**	**1.5**	0.1
	p = 0.6	**p = 0.004**	**p = 0.03**	**[0.3, 2.5]**	[−0.9, 1.0]
duration A	F_1,18_ = 0.7	F_2,30_ = 2.3	F_2,30_ = 1.4	0.2	−1.0
	p = 0.4	p = 0.1	p = 0.3	[−0.7, 1.2]	[−2.0, 0.01]
duration B	F_1,18_ = 0.2	F_2,30_ = 2.0	F_2,30_ = 0.4	−0.3	−0.4
	p = 0.6	p = 0.2	p = 0.6	[−1.3, 0.7]	[−1.4, 0.5]
duration C	F_1,18_ = 0.4	F_2,30_ = 0.9	F_2,30_ = 0.2	0.7	−0.1
	p = 0.6	p = 0.4	p = 0.8	[−0.3, 1.7]	[−1.1, 0.8]
duration pause A-B	F_1,18_ = 1.2	**F**_2,30_ ** = 7.6**	F_2,30_ = 1.0	**1.2**	0.6
	p = 0.3	**p = 0.002**	p = 0.4	**[0.1, 2.2]**	[−0.4, 1.5]
no. of elements in A	F_1,18_ = 0.6	**F**_2,30_ ** = 23.1**	F_2,30_ = 1.1	−**2.6**	−**1.7**
	p = 0.6	**p<0.0001**	p = 0.4	**[**−**3.9,** −**1.3]**	**[**−**2.8,** −**0.6]**
no. of elements in C	F_1,18_ = 0.04	**F**_2,30_ ** = 12.2**	F_2,30_ = 0.9	−**1.9**	−0.8
	p = 0.8	**p<0.0001**	p = 0.4	**[**−**3.0,** −**0.7]**	[−1.8, 0.2]
freq bandwidth A	F_1,18_ = 1.5	F_2,30_ = 1.1	F2,30 = 2.1	0.01	0.7
	p = 0.2	p = 0.4	p = 0.1	[−1.0, 1.0]	[−0.3, 1.6]
max frequency A	F_1,18_ = 1.4	**F**_2,30_ ** = 3.9**	**F**_2,30_ ** = 5.1**	−0.1	**1.4**
	p = 0.3	**p = 0.03**	**p = 0.01**	[−1.0, 0.9]	**[0.3, 2.4]**
freq bandwidth B	F_1,18_ = 3.7	**F**_2,30_ ** = 4.4**	**F**_2,30_ ** = 5.4**	−**1.4**	−0.1
	p = 0.07	**p = 0.02**	**p = 0.009**	**[**−**2.4,** −**0.3]**	[−1.0, 0.9]
max frequency B	**F**_1,18_ ** = 6.6**	**F**_2,30_ ** = 3.6**	F_2,30_ = 2.0	−**1.0**	−0.4
	**p = 0.02**	**p = 0.04**	p = 0.2	**[**−**2.1, 0.0]**	[−1.3, 0.6]
freq bandwidth C	F_1,18_ = 0.1	F_2,30_ = 0.9	F_2,30_ = 0.09	−0.2	0.02
	p = 0.7	p = 0.4	p = 0.9	[−1.2, 0.8]	[−0.9, 1.0]
max frequency C	F_1,18_ = 0.2	F_2,30_ = 2.1	F_2,30_ = 0.2	0.1	0.2
	p = 0.7	p = 0.1	p = 0.8	[−0.9, 1.0]	[−0.7, 1.2]

Context is a within-subjects factor with three levels: before STI (spontaneously sung songs), during STI (playback and decoy present) and after STI (directly after removal of playback and decoy). Treatment is a between-subjects factor with two levels: placebo-implanted vs. blocker-implanted males. To control for repeated measures the ID of each territory owner was included as random intercept. Significant results are highlighted in bold.

**Figure 3:**
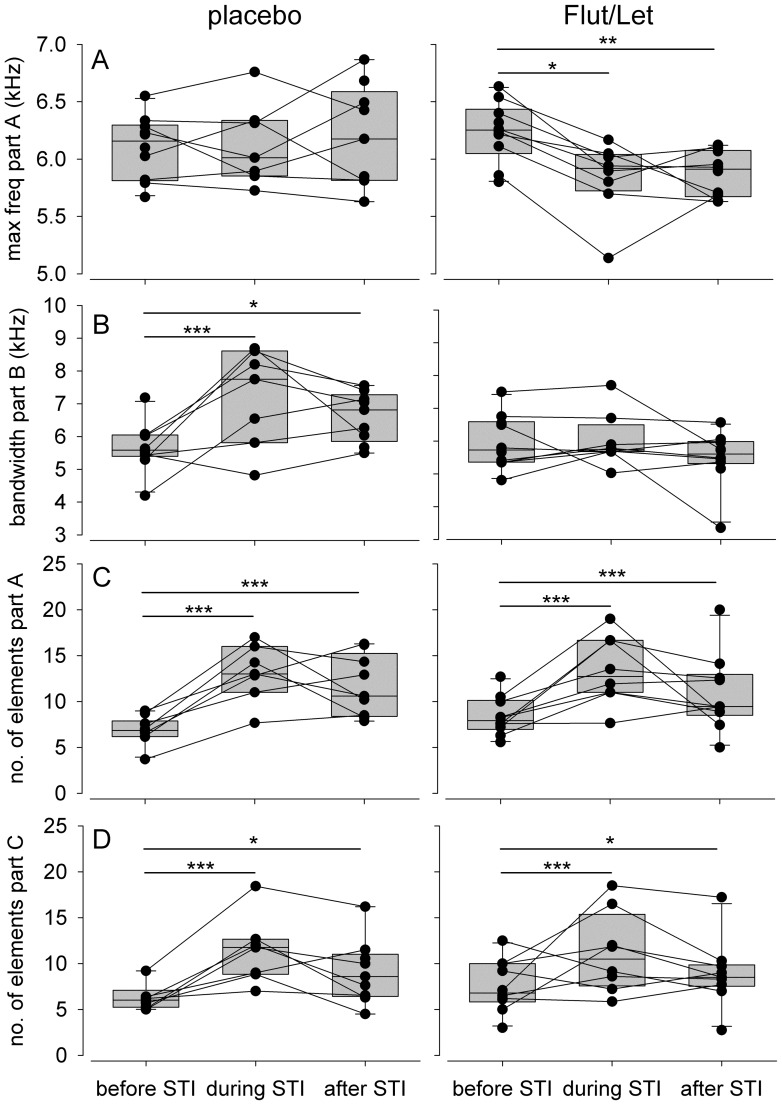
Structural song measures before, during and after the STI. **** Depicted separately for males treated with flutamide and letrozole (‘Flut/Let’, n = 10) and placebo treated males (‘placebo’, n = 10) in spring. Each circle represents one individual male and measurements of the same male are connected by a line. Asterisks indicate significance (* p<0.05, ** p<0.01, *** p<0.001) and are according to a priori set contrasts (before vs. STI and before vs. after the STI).

Both placebo-implanted and Flut/Let-males sang significantly more elements in song parts A and C during and after the STI than before the STI (Table 1, Figs. 3c, d). This element increase resulted from an increase in the number of elements of the trilled phrases of part A or C, respectively (Fig. 1). By definition, part B did not change with respect to this measure because it consisted of one element only (Fig. 1).

Flut/Let-males sang part A with a significantly lower maximum frequency during and after the STI than before the STI. In contrast, the maximum frequency of part A did not change before, during and after the STI in placebo-implanted males (Table 1, Fig. 3a). Both treatment groups sang part B with a significantly higher maximum frequency during the STI than before the STI. Furthermore, the maximum frequency of this part tended to remain high after the STI in placebo-implanted males but not in Flut/Let-males (Table 1). Consequently, placebo-implanted males sang part B with a significantly larger frequency bandwidth during and after the STI than before the STI, while frequency bandwidth of part B did not change in Flut/Let-implanted males (Table 1, Fig. 3b).

Maximum frequency and the frequency bandwidth of part C did not change in response to the STI or Flut/Let-treatment (Table 1).

### Song in Fall

**Figure 4:**
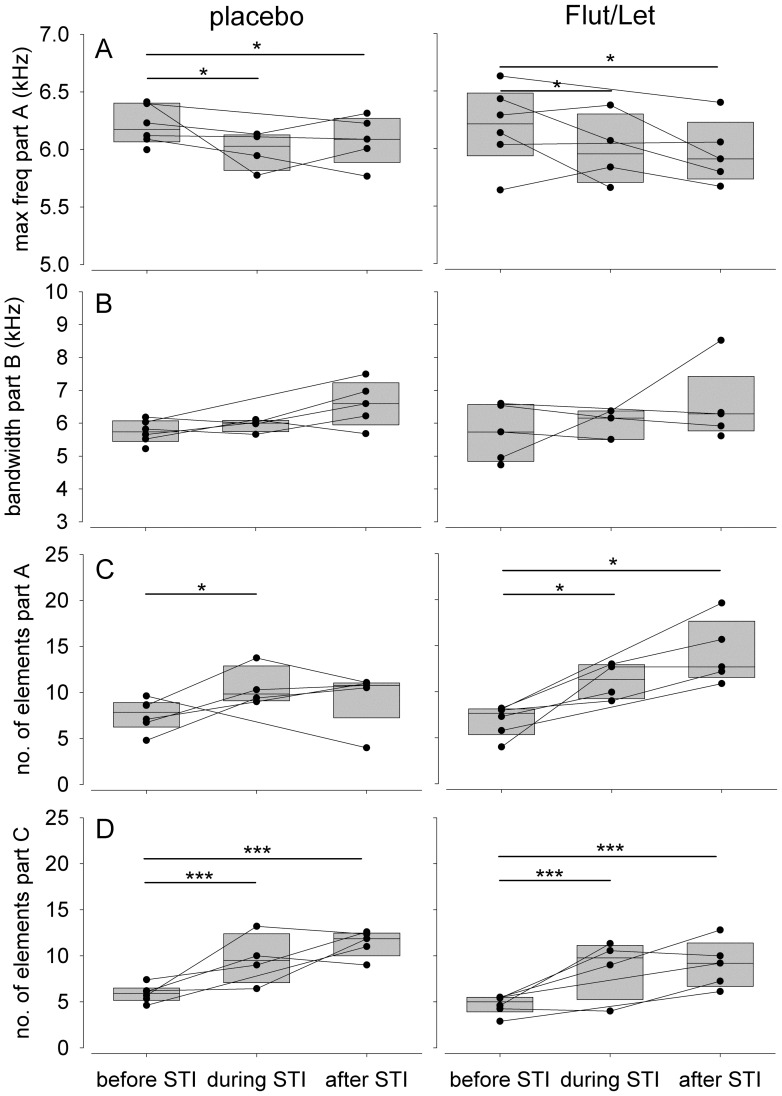
Structural song measures before, during and after the STI. Depicted separately for males treated with flutamide and letrozole (‘Flut/Let’, n = 6) and placebo treated males (‘placebo’, n = 6) in fall. Each circle represents one individual male and measurements of the same male are connected by a line. Asterisks indicate significance (* p<0.05, *** p<0.001) and are according to a priori set contrasts (before vs. STI and before vs. after the STI).

In both treatment groups focal males sang fewer songs during and after the STI than before the experimental challenge (Fig. 2b, Table 2). Males of both treatment groups increased the number of elements in part A (Fig. 4c) and C (Fig. 4d) in response to the experimental challenge while decreasing the maximum frequency of part A (Fig. 4a) and decreasing the frequency bandwidth of part C (Table 2). Males sang part B with a significantly higher maximum frequency in response to the simulated territorial intrusion than during spontaneous song and this did again not significantly differ between placebo and Flut/Let-implanted males (Table 2). However, this effect is not reflected in a higher frequency bandwidth of part B in fall, in contrast to placebo-implanted males in spring (Fig. 4b, Table 2). Furthermore, changes in the frequency bandwidth of part B occur at a far narrower range in fall than in spring (Figs. 3b and 4b). With regard to the effect sizes (Table 2) we suggest to treat the results on frequency measures in fall with caution.

**Table 2:** Linear mixed model results for the effects of context and Flut/Let treatment on song output and structure in fall.

element	treatment	context	interaction	Coheńs d [95%CI]		
				placebo	Flut/Let	
song rate	F_1,10_ = 3.0	**F**_2,18_ ** = 43.1**		**2.3**	**2.4**	
	p = 0.1	**p<0.0001**		**[0.8, 3.8]**	**[0.8, 3.9]**	
song duration	F_1,10_ = 0.02	F_2,18_ = 2.1		0.8	0.1	
	p = 0.9	p = 0.1		[−0.4, 2.9]	[−1.0, 1.2]	
duration A	F_1,10_ = 4.3	F_2,18_ = 3.2		0.3	−1.3	
	p = 0.06	p = 0.06		[−0.8, 1.5]	[−1.3, 1.0]	
duration B	F_1,10_ = 0.01	F_2,18_ = 0.7		0.7	0.05	
	p = 0.9	p = 0.5		[−0.5, 1.8]	[−1.1, 1.2]	
duration C	F_1,10_ = 2.2	F_2,18_ = 3.4		−0.1	−0.7	
	p = 0.2	p = 0.06		[−1.2, 1.0]	[−1.9, 0.5]	
duration pause A-B	F_1,10_ = 0.4	F_2,18_ = 0.1		−0.2	0.6	
	p = 0.5	p = 0.9		[−1.3, 1.0]	[−0.6, 1.7]	
no. of elements in A	F_1,10_ = 2.4	**F**_2,14_ ** = 11.9**	F_2,14_ = 3.8	−**1.6**	−**2.6**	
	p = 0.2	**p<0.001**	p = 0.05	**[**−**3.0,** −**0.1]**	**[**−**4.1,** −**0.9]**	
no. of elements in C	F_1,10_ = 0.2	**F**_2,16_ ** = 26.1**		−**4.2**	−**2.3**	
	p = 0.2	**p<0.0001**		**[**−**6.4,** −**1.9]**	**[**−**3.7,** −**0.7]**	
freq bandwidth A	F_1,10_ = 0.2	F_2,16_ = 2.0		0.6	2.1	
	p = 0.7	p = 0.2		[−0.6, 1.8]	[0.5, 3.6]	
max frequency A	F_1,10_ = 0.08	**F**_2,16_ ** = 3.9**		0.7	0.9	
	p = 0.8	**p = 0.04**		[−0.6, 1.9]	[−0.3, 2.1]	
				**combined: 1.0 [0.1,0.9]**		
freq bandwidth B	F_1,10_ = 0.7	F_2,13_ = 3.2		−1.6		−0.6
	p = 0.4	p = 0.08		[−3.0, −0.2]		[−1.8, 0.6]
max frequency B	F_1,10_ = 1.5	**F**_2,13_ ** = 5.0**		−**1.9**		−0.8
	p = 0.2	**p = 0.02**		**[**−**3.3,** −**0.4]**		[−2.0, 0.5]
freq bandwidth C	F_1,10_ = 0.03	**F_2,16_ = 3.6**		1.0		0.5
	p = 0.9	**p = 0.05**		[−0.3, 2.3]		[−0.7, 1.6]
max frequency C	F_1,10_ = 0.1	F_2,16_ = 2.2		0.8		0.3
	p = 0.7	p = 0.1		[−0.5, 2.9]		[−0.9, 1.4]

Context is a within-subjects factor with three levels: before STI (spontaneously produced songs), during STI (playback and decoy present) and after STI (directly after removal of playback and decoy). Treatment is a between-subjects factor with two levels: placebo-implanted versus blocker-implanted males. Significant results are highlighted in bold.

## Discussion

In this study, we explored the role of testosterone (and its estrogenic metabolites) in modulating song characteristics of black redstarts in a spontaneous and a reactive context both during breeding and non-breeding. Territorial males of both treatment groups and in both seasons did change structural song parameters in an aggressive context. In spring, both treatment groups increased the number of elements sung in parts of their song in response to the STI. However, Flut/Let males decreased the maximum acoustic frequency of one song part in response to the STI, while placebo-implanted males kept this acoustic measure constant throughout the challenge. Furthermore, placebo-implanted males sang the atonal part of their song with a broader frequency range. In contrast to Flut/Let males, placebo-implanted males increased signal density by singing shorter songs with shorter pauses between song parts in the STI. In summary, these results provide a good example of the activational role of testosterone not only on song activity in general, but also on the specific singing style depending on the context.

The results of this study indicate that song sung during a territorial encounter is of higher competitive value than song sung in an undisturbed situation and may, therefore, convey information about the motivation or quality of the territory holder. During simulated intrusions in fall, when testosterone levels are naturally low in this species, males of both treatment groups sang similar to Flut/Let-implanted males during breeding. We conclude that these changes in song in response to a simulated territorial intruder were influenced by the Flut/Let treatment and by season: structural changes in song were less pronounced in Flut/Let males and in all males during non-breeding in fall compared to placebo-implanted males in spring.

### Song Modulation during Territorial Challenges

Black redstarts of both treatment groups in spring sang more elements in parts A and C and placebo-implanted birds increased the frequency bandwidth of part B when a simulated rival intruded the territory. Additionally, Flut/Let males decreased the maximum frequency of part A. These structural song parameters have been suggested to be physically challenging in other species (reviewed in [16]). Also, with regard to trilled parts, it has been suggested previously that the production of repeated (trilled) syllables with a high frequency bandwidth is challenging (reviewed in [16]). For example, in swamp sparrows, male age, size, and early developmental conditions correlated with these song characteristics, and can therefore serve as honest signals of male quality [14], [56], [5]. Females of some species prefer songs sung with a high trill rate and broad frequency bandwidth [12], [57]. Furthermore, swamp sparrows increase both trill rate and frequency bandwidth in response to simulated territorial intruders [10].

Even though songs of control males were shorter during the STI than before (which might occur counter-intuitive at first, since usually birds increase song output when challenged), this resulted in a higher signal density. Increasing the signal density by changing the song output in an aggressive context seems to be a common strategy among bird species (e.g. [30], [58]). In our study on black redstarts, this increase was realized by a shortening of pauses between song parts.

### Treatment and Season Affect Song Modulation during Territorial Challenges

Although all males (regardless of treatment and season) changed their song in the aggressive context, Flut/Let males in spring and all males challenged during non-breeding in fall did so to a lesser extent than placebo males during breeding in spring. The changes that we find to be inhibited by the Flut/Let-treatment in spring (i.e. maximum frequency of part A and frequency bandwidth of part B) are similar to the parameters Cucco and Malacarne (1999, [47]) found to be characteristic for adult males song as opposed to yearling males’ song. These parallels in acoustic features that differ between age-groups [47] and males of different hormonal status (our study) deserve further consideration. Yearlings as well as males with low testosterone levels might fail to produce challenging acoustic features due to lack of experience. Considering that adult male black redstarts (singing ‘mature song’) in general have a higher reproductive success than yearlings [48], [49], we assume that this mature song is indicating a better quality and our Flut/Let-implanted males failed to produce this ‘mature song’. Thus, context-dependent changes in song structure may indeed reveal information about the quality of the producer.

In Flut/Let-implanted males during spring and all males during fall the increase in the number of elements in part A was associated with a decrease in its maximum frequency. Therefore, Flut/Let-males in spring and all males in fall tended to sing this song part with a lower frequency bandwidth during a challenge than during spontaneous song. This might be interpreted as a failure to increase the number of elements and maintain the frequency at the same time in terms of a performance constraint, or alternatively, that Flut/Let-implanted males invested less into the production of these signals than did placebo-implanted birds in spring. Considering that territorial behaviors other than song were not affected by a Flut/Let-treatment in spring (Apfelbeck et al., under revision) it is likely that motivational differences can not exclusively account for our results.

In addition, in contrast to placebo-implanted males Flut/Let males did not increase the frequency bandwidth of song part B. Part B consists only of a single noisy song element. Noisy elements are characterized as atonal, non-harmonic sounds occupying a range of frequencies (Fig. 1). There are good reasons to assume that such atonal song elements are not produced by the syrinx but by modulating the airflow in the vocal tract (reviewed in [59]). Accordingly, placebo-implanted birds may sing with a higher air pressure and thus louder than Flut/Let-males. As a consequence, a broader range of frequencies is “broadcasted” in placebo-implanted males than in Flut/Let-males. Alternatively, a broader frequency range might be achieved by an increase in beak opening (e.g. [60]). In barn swallows, the song characteristics of a similar harsh or noisy element, the rattle, were correlated with testosterone concentration [30]. Changing the acoustic properties of such elements within limits may be interpreted as a way to increase their signal value as described in the framework of index signals.

Our results obtained in breeding and non-breeding males suggest that some, though not all song response measures in an aggressive context are mediated by testosterone or estradiol. Blocking these hormones particularly affected structural song measures. This may indicate that testosterone represents an underlying mechanism allowing the modification of ‘index signals’ such as trill rate or frequency measures. Similar results were recently reported for singing mice [61], [62] and may thus reflect a general mechanism in vertebrates. In birds, such a modification within limits may be achieved for example by modifying properties of the syrinx, an organ that is sensitive to testosterone and estradiol [63]–[65], or the beak muscles [60]. In addition, the neuronal coordination of singing might be affected by changes in testosterone levels, too (reviewed in [66]). Studies on male Gambel’s white-crowned sparrows (*Zonotrichia leucophrys Gambelii*) did provide evidence that decreasing or increasing testosterone levels in the brain can affect the nuclei of the song control system within very few days or even hours [67], [68]. Furthermore, in some species testosterone levels rise during territorial disputes (reviewed in [69], [70] and this increase in testosterone may affect the behavior and persistence of the male during or after the challenge [71], [72]. However, black redstarts do not show such a short-term increase in testosterone levels during territorial intrusions [51], [73]. Thus, any effects of testosterone on song and territorial behavior probably take place as a consequence of the increase in testosterone at the beginning of the breeding season (i.e. an activational effect).

Furthermore, these context-dependent changes in song may be regulated by aromatase activity in the pre-optic area [40], [74] or changes in androgen and estrogen sensitivity in the song control nucleus HVC [75] because redstarts show a higher expression of aromatase mRNA in the pre-optic area in spring than in fall, but no seasonal change in HVC size (Apfelbeck et al., under review).

### Conclusions

Our study demonstrates that blocking the actions of testosterone affected both song output and structural song measures of black redstarts during competitive situations during the breeding season, but not outside the breeding season. We conclude that testosterone may affect both the signal parameters indicating the motivation and/or the ability to engage in competitive interactions such as territorial disputes. This might be achieved by effects of testosterone on the neuronal and physiological capabilities to produce certain song elements depending on the behavioral context. This very nicely illustrates that hormones can change the likelihood of a behavior, often in a context-dependent manner [76].
