# Correction: Testosterone Affects Song Modulation during Simulated Territorial Intrusions in Male Black Redstarts (*Phoenicurus ochruros*)

**DOI:** 10.1371/annotation/89b74b56-3f48-4ee2-9da1-699b3fdea564

**Published:** 2012-12-28

**Authors:** Beate Apfelbeck, Sarah Kiefer, Kim G. Mortega, Wolfgang Goymann, Silke Kipper

There was an error in the Competing Interests statement. The correct Competing Interests statement is as follows: I have read the journal's policy and have the following competing interests: Novartis provided us with the aromatase inhibitor “letrozole” for our experiments. This does not alter our adherence to all the PLOS ONE policies on sharing data and materials.

